# Impact of Layer Materials, Their Thicknesses, and Their Reflectivities on Emission Color and NVIS Compatibility in OLED Devices for Avionic Display Applications

**DOI:** 10.3390/mi16020191

**Published:** 2025-02-07

**Authors:** Esin Uçar, Alper Ülkü, Halil Mert Kaya, Ramis Berkay Serin, Rifat Kaçar, Ahmet Yavuz Oral, Ebru Menşur

**Affiliations:** 1 Department of Materials Science and Engineering, Gebze Technical University, Kocaeli 41400, Türkiye; aulku@aselsan.com (A.Ü.); rbserin@aselsan.com (R.B.S.); aoral@gtu.edu.tr (A.Y.O.); ebrualkoy@gtu.edu.tr (E.M.); 2 ASELSAN Avionics and Guidance Systems Business Sector, Avionics Design Directorate, Ankara 06750, Türkiye; hmertkaya@aselsan.com (H.M.K.); rifatkacar@aselsan.com (R.K.)

**Keywords:** OLED, NVIS, reflectivity, HIL, HTL, ETL, luminance, current efficiency, SETFOS, LightTools

## Abstract

Organic Light Emitting Diode (OLED) technology is preferred in modern display applications due to its superior efficiency, color quality, and flexibility. It also carries a high potential of applicability in military displays where emission color tuning is required for MIL-STD-3009 Night Vision Imaging Systems (NVISs), as compatibility is critical. Herein, we report the effects of different OLED device layer materials and thicknesses such as the hole injection layer (HIL), hole transport layer (HTL), and electron transport layer (ETL) on the color coordinates, luminance, and efficiency of OLED devices designed for night vision (NVIS) compatibility. In this study, simulation tools like SETFOS^®^ (Semi-conducting Emissive Thin Film Optics Simulator), MATLAB^®^, and LightTools^®^ (Illumination Design Software) were used to verify and validate the luminance, luminance efficiency, and chromaticity coordinates of the proposed NVIS-OLED devices. We modeled the OLED device using SETFOS^®^, then the selection of materials for each layer for an optimal electron–hole balance was performed in the same tool. The effective reflectivity of multiple OLED layers was determined in MATLAB^®^ in addition to an optimal device efficiency calculation in SETFOS^®^. The optical validation of output luminance and luminous efficiency was performed in LightTools^®^. Through a series of simulations for a green-emitting OLED device, we observed significant shifts in color coordinates, particularly towards the yellow spectrum, when the ETL materials and their thicknesses varied between 1 nm and 200 nm, whereas a change in the thickness of the HIL and HTL materials had a negligible impact on the color coordinates. While the critical role of ETL in color tuning and the emission characteristics of OLEDs is highlighted, our results also suggested a degree of flexibility in material selection for the HIL and HTL, as they minimally affected the color coordinates of emission. We validated via a combination of SETFOS^®^, MATLAB^®^, and LightTools^®^ that when the ETL (3TPYMB) material thickness is optimized to 51 nm, the cathode reflectivity via the ETL-EIL stack became the minimum enabling output luminance of 3470 cd/m2 through our emissive layer within the Glass/ITO/MoO_3_/TAPC/(CBP:Ir(ppy)_3_)/3TPYMB/LiF/Aluminum OLED stack architecture, also yielding 34.73 cd/A of current efficiency under 10 mA/cm^2^ of current density. We infer that when stack layer thicknesses are optimized with respect to their reflectivity properties, better performances are achieved.

## 1. Introduction

Since the early 2000s, LCD technology has dominated the display market due to its widespread availability and reliability; however, the lack of self-emissivity and the need for a backlight unit increases panel thickness and reduces flexibility [1,2,3,4]. With the development of self-emitting organic light-emitting diode (OLED) technology, OLED displays have gained popularity and emerged as strong alternatives to LCDs. OLEDs offer notable advantages, such as high contrast ratios, low power consumption, self-emissivity, a wide color gamut, enhanced viewing angles, deeper blacks, and flexible form factors [5,6,7,8,9,10,11].

Display technologies play a critical role in military avionic applications by enabling pilots and aircrews to quickly and easily access essential information. Military operations often occur during nighttime or under low-light conditions, where night vision imaging systems are vital. Pilots command avionic systems while using NVIS goggles under cockpit lighting. As a result, all avionic equipment, including displays, must comply with the NVIS compatibility requirements for nighttime [12,13,14,15,16]. To maintain NVIS compatibility, avionic display colors must strictly remain within distinct circles on the chromaticity diagram. The NVIS color coordinates are specific values that define how colors appear under night vision conditions. These coordinates ensure that displays are optimized for visibility and performance in low-light environments, making them suitable for military and aviation applications. For each color (green, red, and yellow), circle boundaries are defined by the MIL-STD-3009 standard [17,18,19,20]. NVIS compatibility significantly influences the performance of OLED displays across various applications by necessitating specific design and material considerations to ensure both operational effectiveness and compliance with night vision technologies.

**Figure 1:**
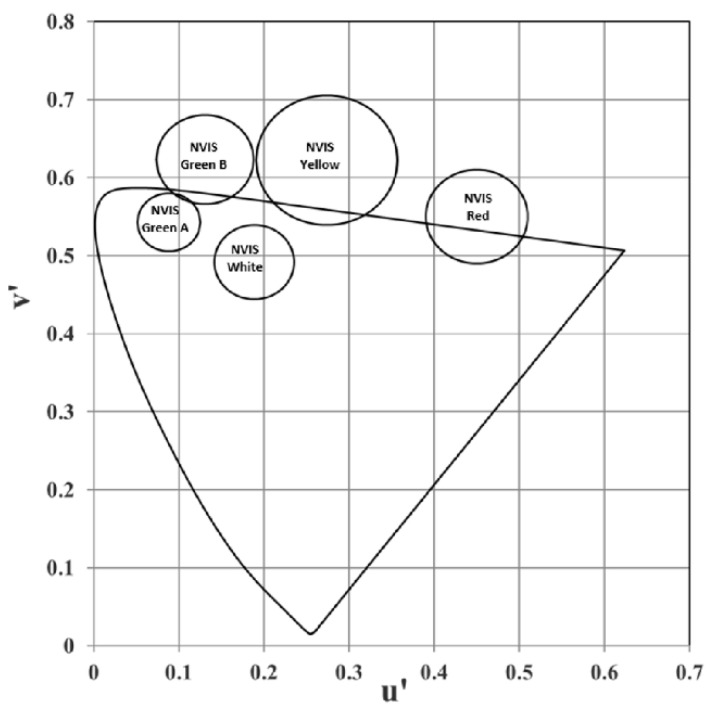
NVIS compatible color coordinates per CIE 1976.

For OLED display devices to be used and be NVIS compatible, the NVIS chromaticity coordinates of red, green, yellow and white colors have to be within the ranges shown in both in Figure 1 and Table 1 as stated in MIL-STD-3009 [17,21]. 

Color is represented as a two-dimensional quantity. Initially, the CIE 1931 chromaticity diagram was introduced, where each color is represented by (x, y) coordinates. However, due to perceptual non-uniformities in this system, the CIE 1976 (u′, v′) uniform chromaticity scale (UCS) diagram was later adopted. This system provides a more uniform representation of color differences as perceived by the human eye. The transformation from (x, y) to (u′, v′) coordinates is given by the following [22]:

(1) $$u^{\prime }=\frac{4x}{-2x+12y+3}\;\;v^{\prime }=\frac{9y}{-2x+12y+3}$$

In NVIS (Night Vision Imaging System) tests, u′ and v′ coordinates are used to define the acceptable range of colors to ensure compatibility with night vision devices. Additionally, the parameter *r* refers to the radius used to draw the boundaries of the NVIS-compliant regions for specific colors, such as red and green, on the CIE 1976 (*u*′, *v*′) diagram. These circular boundaries help verify if a display’s emission falls within the specified limits for NVIS compliance, as defined in MIL-STD 3009. The color coordinates in Table 1 represent the color circles specified in MIL-STD 3009 that a military display is required to meet.

OLED displays face significant technical challenges, especially when applied to aerospace environments. These challenges include susceptibility to environmental factors such as humidity, harsh temperatures, solar radiation, and vibration. Such conditions can degrade the OLED materials, affecting the longevity and reliability of the display systems crucial for avionics applications [22,23]. Although advancements in polymer light-emitting diodes (PLEDs) and the incorporation of nanoparticles have been made to increase thermal stability and electroluminescent efficiency, these solutions are still in the developmental stage and have not fully addressed the longevity and robustness required for avionics displays [4,24].

Military missions often require operations during nighttime or in low-light conditions thus pilots use night vision imaging systems on avionic platforms, and they command avionic systems while using NVIS goggles under cockpit lighting. Because of this, NVIS compatibility is required for all avionic equipment, including displays during night operations. The transition from the night vision standards MIL-L-85762 to MIL-STD-3009 [17,21] highlighted the importance of maintaining essential interface characteristics for NVIS compatibility, emphasizing the need for displays to meet stringent standards without detailed design prescriptions [9,25,26].

In the realm of OLED displays, achieving NVIS compatibility involves several innovative approaches. Dunn [27] introduced a method that customizes a polaroid film to correct the chroma of the display and then applies a filter glass designed to cut off specific spectral energy distributions, thereby aligning the OLED’s emission spectrum with NVIS operational bands. In an OLED device, the light-emitting layer (EML) primarily determines the emission color. However, studies show that other layers, including the hole injection layer (HIL), hole transport layer (HTL), and electron transport layer (ETL) can also affect the final color output. These layers influence the charge balance, recombination efficiency, and optical properties, potentially altering the emission characteristics and performance of the final OLED device [10,27,28,29,30,31,32,33,34].

The integration of OLED displays in electronic devices significantly enhances NVIS compatibility due to several key factors. OLED displays exhibit characteristics that make them highly compatible and beneficial for night vision applications. Their large contrast ratios and wide color gamut enable clear and distinct image presentation in low-light conditions, which is crucial for NVIS compatibility [35,36,37]. These features also contribute to the reduction of the overall power consumption, a critical factor for devices used in night operations [38,39,40].

The thickness and materials used in OLEDs significantly impact the emitted color. Varying the thickness of the emitting layer can optimize device performance [41,42], with thinner layers often leading to lower turn-on voltages and a higher luminance. The different materials in the OLED layers play crucial roles in color emission [43,44]. For instance, using blue and red emitting materials like Zn(HPB)_2_ and Ir-complexes can result in white OLEDs when combined with a green emitting layer. Additionally, the choice of materials like Rubrene, FIrPic, and MCP can influence the color temperature of the emitted light, shifting from warm white to cold white with changes in driving voltage [20]. The thickness of the various layers in organic light-emitting diodes (OLEDs) significantly impacts their luminance efficiency, as demonstrated by multiple studies [30,44,45,46,47,48,49,50]. A comprehensive simulation model revealed that a 50 nm thick emitting layer (EML) in a trilayer OLED structure resulted in a remarkable luminance of 10,000 cd/m^2^, indicating the importance of EML thickness in device performance [51,52]. The study of blue fluorescent and yellow phosphorescent OLEDs highlighted that increasing the electron-transporting matrix thickness from 20 to 80 nm improved current efficiency from 15.7 to 31.7 cd/A, while thicker EMLs in blue devices reduced efficiency due to hindered hole transfer [53]. Another study examined the effect of varying the thickness of the electron-injection layer (EIL) composed of Cs_2_CO_3_; in OLEDs. The critical finding was that a 1.0 nm thick EIL significantly improved luminance and external quantum efficiency by approximately 600% and 500%, respectively, compared to the devices without an EIL. This enhancement was attributed to the Cs_2_CO_3_; in the EIL reducing the potential barrier at the cathode by 0.08 eV, which facilitated electron injection as well as hole movement blocking, and contributed to a higher recombination rate, further boosting the overall efficiency of the OLED device [54]. The optimal thickness of a copper iodide (CuI) hole injection layer (HIL) was found to be 12 nm, which maximized luminescence current efficiency [55]. As the HIL is responsible for injecting holes into the organic layers from the anode, it helps to enhance the injection of holes at lower voltages, improving the overall efficiency and lifetime of the OLED device. Molybdenum Trioxide (MoO_3_), Copper Phthalocyanine (CuPc), N,N′-Bis(naphthalen-1-yl)-N,N′-bis(phenyl)benzidine (α-NPD), and 1,4,5,8,9,11-Hexaazatriphenylenehexacarbonitrile (HATCN) are used for the hole injection layer (HIL) in organic light-emitting diodes (OLEDs), as they play a crucial role in enhancing the efficiency and stability of these devices. MoO_3_ is often used due to its ability to facilitate an efficient hole injection, which is critical for reducing the operating voltage and improving device performance [56]. CuPc, particularly in its nanocrystalline form, has been shown to significantly improve the power efficiency and external quantum efficiency of OLEDs compared to traditional materials like PEDOT:PSS, due to its lower hole injection barrier and superior mobility [57]. The use of α-NPD and HATCN as HIL materials is also well documented, contributing to the improved charge balance and reduced efficiency roll-off, highly preferable for display applications [58]. These materials, through their unique properties, help in achieving a lower voltage operation and the enhanced lifetime of OLEDs by helping better hole injection and transport, thus making them integral to the development of high-performance OLEDs [59]. Due to their highest performances, our candidate HIL selection reflected the current state-of-the-art, as shown in the “HIL” column in Table 2. 

After the holes are injected, the HTL enables the movement of these holes towards the EML. 1,1-Bis[4-(di-p-tolylamino) phenyl] cyclohexane (TAPC), Tris(4-(9H-carbazol-9-yl) phenyl) amine (TCTA), Tris(9-phenylcarbazol-3-yl) benzene (TrisPCZ), N,N′-Bis(naphthalen-1-yl)-N,N′-bis(phenyl)benzidine (NPB), 3,3′-Bis(N-carbazolyl)-1,1′-biphenyl (mCBP), 1,3-Bis(N-carbazolyl) benzene (mCP), 4,4′,4″-Tris[3-methylphenyl(phenyl)amino] triphenylamine (m-MTDATA), 2,2′,7,7′-Tetrakis [N,N-di(4-methoxyphenyl) amino]-9,9′-spirobifluorene (SpiroOMeTAD), 4,4′-Bis(N-carbazolyl)-1,1′-biphenyl (CBP), and 4,4′,4″-Tris(N-(naphthalen-2-yl)-N-phenylamino) triphenylamine (2-TNATA) are common materials for the HTL. Materials have been identified as effective HTLs, including 1,1-Bis[4-(di-p-tolylamino) phenyl] cyclohexane (TAPC), Tris(4-(9H-carbazol-9-yl) phenyl) amine (TCTA), and others. These materials are chosen for their ability to enhance hole injection and transport, critical for device efficiency. The use of cross-linkable HTL materials has been shown to improve device efficiency significantly by perfecting the interface between the HTL and EML, as demonstrated in green phosphorescent OLEDs where a spin cleaning process was employed to enhance performance [60]. The use of p-doped HTLs in multilayer electroluminescent devices has also been shown to lower operating voltages and improve efficiency [61]. Thermally cross-linkable HTLs have been developed to match energy levels with the anode and light-emitting polymers, facilitating efficient hole injection and transport in multilayer devices [61,62,63,64]. These advancements highlight the critical role of HTLs in improving the performance of OLEDs and related devices, with ongoing research focusing on material innovation and structural optimization to further enhance device efficiency and longevity. Due to their highest performances, our candidate HTL selection reflected the current state-of-the-art, as shown in the “HTL” column in Table 2. 

The ETL is used to ease the movement of electrons and to block the movement of holes, ensuring that the electrons and holes meet and recombine in the EML to produce light. Tris[3-(3-pyridyl) mesityl] borane (3TPYMB), 1,3,5-Tris(N-phenylbenzimidazol-2-yl) benzene (TPBi), 2,9-Dimethyl-4,7-diphenyl-1,10-phenanthroline (Bathocuproine) (BCP), Tris[4-(pyridin-3-yl) phenyl] amine (TMPYPB), 4,7-Diphenyl-1,10-phenanthroline (Bathophenanthroline) (BPHEN), Bis(2-methyl-8-hydroxyquinolinato) aluminum (III) (Balq), Bis-4,6-(3,5-di(pyridin-3-yl) phenyl)-2-methylpyrimidine (B4PYMPM), Bis-4,6-(3-(pyridin-3-yl) phenyl)-2-methylpyrimidine (B3PYMPM), and Tris(8-hydroxyquinolinato) aluminum (III) (Alq_3_) are common materials for the ETL described in the literature. Various materials have been explored for their effectiveness as ETLs, each offering unique advantages. Tris(8-hydroxyquinolinato) aluminum (Alq_3_) is a well-studied ETL material known for its electron transport capabilities and has been used in numerous studies to enhance device performance. The inclusion of dual electron-transport layers (d-ETL) with hole-blocking functions, such as Bpy-OXD, has been shown to improve chromaticity and electron injection, leading to higher power efficiency and a reduced driving voltage [65]. Additionally, novel compounds like aluminum chelates, such as tris(9-oxidophenalenone) aluminum, have been developed to offer improved thermal stability and comparable efficiencies, further enhancing the performance of OLEDs [66]. The use of low molecular weight and polymeric heterocyclics, characterized by high ionization potential values, also contributes to effective electron injection and hole-blocking, reducing interface barriers and enhancing device efficiency. The integration of materials with hole-blocking capabilities into the ETL, as seen in some innovative device structures, simplifies fabrication and increases efficiency [67,68].

These findings collectively underscore the critical role of layer material thickness in enhancing luminance efficiency, with each layer’s optimal thickness contributing to the overall device performance through an improved charge carrier balance and reduced leakage currents.

Our research comes to stage as the most comprehensive simulation work including 35 different stacked combinations of OLED materials, examining the effects of the HIL, HTL, and ETL materials and thicknesses on color coordinates and also aiming especially to achieve high luminous efficiency and NVIS compatibility. It will be the first paper to analyze and validate the performance of NVIS-OLED devices in both SETFOS and LightTools simultaneously. We call the proposed device “NVIS-OLED”. The NVIS-OLED stack is simulated in performance with 10 different ETL, 10 different HTL, and 4 different HIL material combinations, varying their thicknesses within the 1–200 nm range. This manuscript presents the simulation results obtained using SETFOS^®^ and LightTools^®^ for the proposed NVIS-OLED structure. In our study, the layer with the greatest effect on the color coordinates of an OLED device architecture was determined. Then, the effects of layer thickness and the material refractive index on both the color coordinates and the reflection profile of the device were investigated. In Section 2, the NVIS-OLED device structure, its geometry, and the EIL, ETL and HTL combinations are introduced. In Section 3, details of our mathematical methods for NVIS-OLED stack modeling, the relationships between layer thickness and the reflection properties of the OLED device, the simulation tools used, and the choice of performance metrics are presented. Complete simulation work is carried out using three different analysis programs: (i) SETFOS^®^ (ver. 5.1), the primary tool for the optical and electrical analysis of organic material structures [68,69,70]; (ii) MATLAB^®^ (ver. R2023b) the essential tool for our optical calculations [71,72,73,74]; and (iii) LightTools^®^ (ver. 9.1.1), analysis software for optical ray tracing and the transmissivity/reflectivity analysis of our proposed NVIS-OLED structure [75,76,77]. Finally, our simulation results are discussed, and the conclusion are reported in Section 4. Additional supplementary information on the mathematical calculations is presented in Supplementary File S1 and complete results for 35 simulations are reported in Supplementary File S2.

## 2. Materials and Methods

Our methods, mathematical derivations, modelling, and evaluation presented herein are staged within the following main steps: First, we modelled the OLED device and selected materials for each layer for optimal electron–hole balance in SETFOS^®^. Followed by the calculations of optimal device efficiency in SETFOS^®^ and the effective reflectivity of multiple OLED layers in MATLAB^®^. Finally, optical validation for output luminance and luminous efficiency was performed in LightTools^®^. Here, we lay the details of each stage as follows.

### 2.1. Modelling of OLED Device Stack

**Figure 2:**
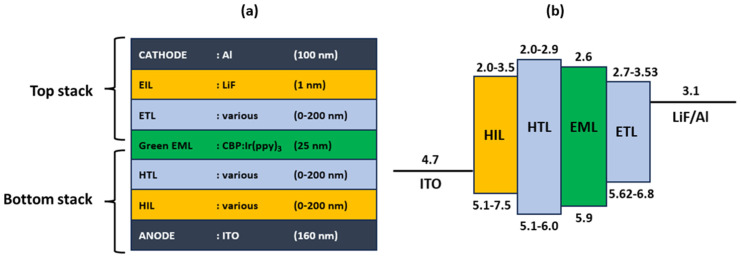
(**a**) Proposed OLED device stack for NVIS-OLED, and (**b**) related energy band gap diagram (energy levels in eV). Various materials for HIL, HTL, and ETL listed in Table 2.

The simulated OLED device architecture and layer thicknesses are given in Figure 2. This structure includes ITO and aluminum as the anode and cathode electrodes, respectively, LiF as the electron hole injection layer, green colored CBP:Ir(ppy)_3_ as the emitting layer. The various materials in Table 2, which are commonly used for these layers in the literature, were chosen and used as the hole injection layer (HIL), hole transport layer (HTL), and electron transport layer (ETL). 

### 2.2. Selection of Materials for Each Layer for Optimal Electron–Hole Balance

**Figure 3:**
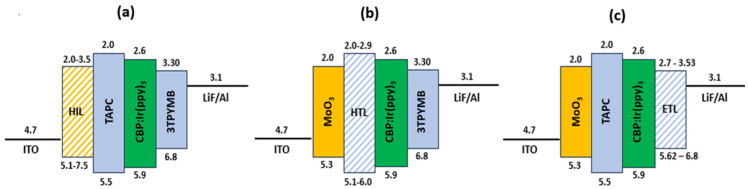
OLED device models by varying (**a**) HILs, (**b**) HTLs, and (**c**) ETLs keeping emissive layer, anode, and cathode constant.

Due to the facts and performances presented in Section 1, our candidate ETL selection reflected the current state-of-the-art, as shown in the “ETL” column in Table 2. ITO has a relatively high work function (around 4.7 eV), which helps inject holes (positive charge carriers) into the adjacent hole injection layer. The HIL further facilitates hole injection into the HTL by lowering the energy barrier, making it easier for holes to flow toward the emission layer CBP:Ir(ppy)_3_. On the opposite side, the cathode (LiF/Al) is connected to the negative terminal of the applied voltage, providing negative charge carriers. The LiF/Al layer acts as an electron injection layer as Lithium Fluoride (LiF) lowers the work function of aluminum, enhancing electron injection from the cathode into the ETL and ensuring electrons are efficiently transported toward the emission layer. When the HIL, HTL and ETL materials are interchanged within the same OLED device, model combinations become as given in Figure 3. In Figure 3a, MoO_3_, CuPc, α-NPD, and HATCN were used interchangeably as HIL materials. The other materials for the stack are TAPC, CBP:Ir(ppy)_3_, 3TPYMB, LiF, and Al. In Figure 3b, TAPC, TCTA, TrisPCZ, NPB, mCBP, mCP, m-MTDATA, SpiroOMeTAD, CBP, and 2-TNATA were used interchangeably as HTL materials. The remaining materials for this device are MoO_3_, CBP:Ir(ppy)_3_, 3TPYMB, LiF, and Al. In Figure 3c, 3TPYMB, TPBi, BCP, 3TMPYPB, BPhen, Balq, B4PYMPM, B3PYMPM, Alq_3_, and Alq were used interchangeably as ETL materials. The remaining materials for this device are MoO_3_, TAPC, CBP:Ir(ppy)_3_, LiF, and Al. In all three devices, LiF and Al thicknesses were kept at 1 nm and 100 nm, respectively.

Materials for the anode, EML, EIL, and cathode of our NVIS-OLED were kept as ITO, CBP: Ir(ppy)_3_, LiF, and aluminum, respectively, throughout our simulations. All of the mentioned materials are concisely listed with their abbreviations in Table 3.

### 2.3. Calculation of Effective Reflectivity of Multiple OLED Layers

The thin film interference theory and Fresnel equations [78] were utilized to investigate the mathematical connection between ETL thickness and reflectance in OLED devices by using MATLAB^®^. Changing the ETL thickness changed the reflectance for a higher luminous efficiency. To demonstrate how accurate the approximations were, LightTools^®^ was utilized to perform optical simulations of the device.

In MATLAB^®^, by using the transfer matrix method [78,79], reflectance values were calculated based on the ETL thickness in the constructed thin-film structure. The thin-film layer structure was then transferred to the simulation environment in LightTools^®^ to examine the impact on the optical parameters. These steps allowed for a comprehensive analysis, combining MATLAB’s theoretical approach with LightTools^®^ practical simulation, to assess how changes in ETL thickness influence reflectance and other optical characteristics.

The mathematical method used to lay out how the thickness of the ETL affects the reflectance in OLED devices is included in this paper. The system is modeled, and the luminous efficiency is improved using Fresnel equations and the thin film interference theory [77,80,81,82]. Mathematical background details are given in Supplementary File S1.

An overview of the purpose and importance of examining reflectance in OLED devices using ETL thickness is given in this chapter. Display technology depends on refining luminous efficiency, and understanding reflectance is essential to that optimization.

## 3. Results and Discussions

### 3.1. SETFOS Simulation Results: Impact of Layers on OLED Characteristics

This section presents the results from the SETFOS simulations, focusing on how various OLED layer (HIL, HTL, ETL) materials and thicknesses influence color coordinates.

#### 3.1.1. The Effect of OLED Layers on Color Coordinates

**Figure 4:**
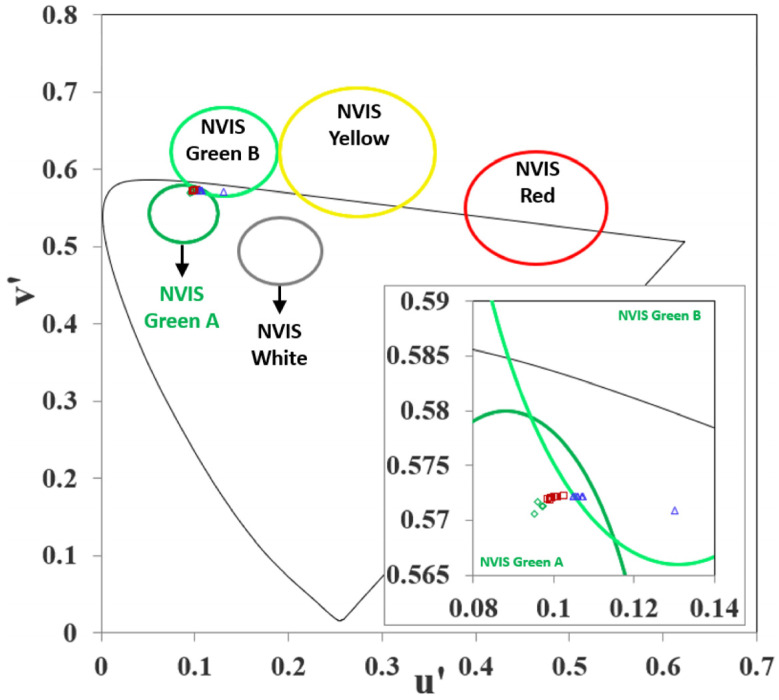
(**a**) CIE 1976 Chromaticity diagrams for different HIL (green diamond), HTL (red square), and ETL (blue triangle) materials and (**b**) inset: zoomed for colorful data points.

In the simulations, the color coordinates of the OLED devices in Figure 3a–c created with different layer materials were determined (Table 4 and Figure 4).

Figure 4 presents the chromaticity diagram, illustrating the (u′, v′) color coordinates for different OLED configurations. The diagram shows the impact of varying color coordinates with different OLED layer materials and the influence of these layers on the emission color. The light and dark green circles represent the NVIS Green A and B compliance regions, respectively, the yellow circle represents the NVIS Yellow compliance region, and the red circle represents the NVIS Red compliance region, within which the related color coordinate needs to inlay. The green diamonds, red squares, and blue triangles, which correspond to specific OLED configurations, are consistently located within or near these compliance regions. These colorful data points highlight the shift in color coordinates as layer materials are altered. These shifts indicate the sensitivity of the emission color to changes in the OLED layer materials. The data points indicate that the materials share a close range of color coordinates within the visible spectrum, as evidenced by their clustering around specific u′-v′ values.

The simulations revealed that variations in the materials used for the HIL and HTL had a minimal impact on the color coordinates of the OLED devices. The color coordinates remained relatively stable, indicating that the choice of material for these layers does not significantly influence the emission spectrum of the OLED. This finding suggests that while the HIL and HTL are crucial for device efficiency and stability, their impact on color tuning is limited. Conversely, a significant shift in color coordinates was observed when different materials were used for the ETL. The color shift ranged predominantly towards the green–yellow spectrum. This pronounced change can be attributed to the alterations in the electron injection and transport properties, which directly affect the recombination zone and consequently the emission spectrum.

**Figure 5:**
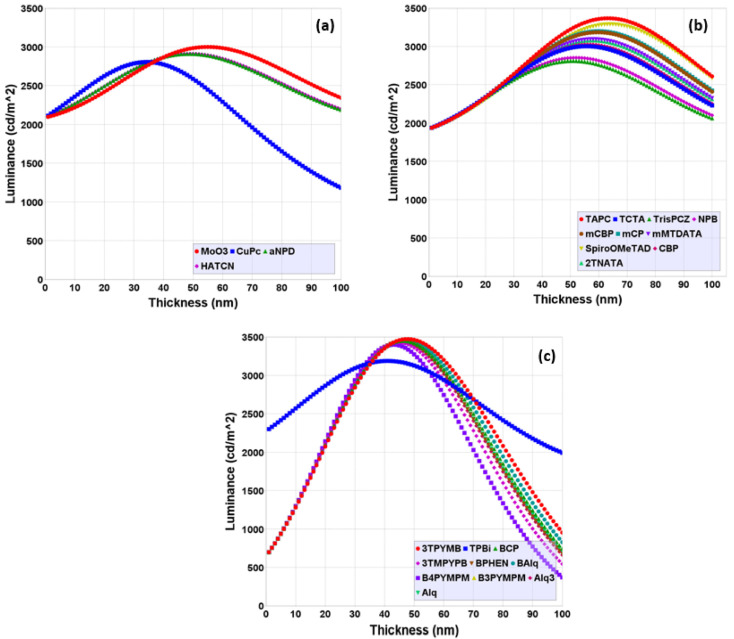
Luminance thickness plots for (**a**) HIL, (**b**) HTL, and (**c**) ETL materials.

The effects of the thickness of the layer materials, the HIL, HTL, and ETL, on the luminance of the NVIS-OLED device are examined in Figure 5. The highest luminance out of the different simulated devices was selected for thickness experiments. The Luminance—HIL-HTL-ETL layer thickness plot (Figure 5a, Figure 5b and  Figure5c, respectively) was used for this selection.

Figure 5 illustrates the luminance characteristics of different thickness values for the HIL, HTL, and ETL materials of Table 2, with luminance generally increasing with thickness up to a peak before gradually decreasing. This behavior suggests the best material and its thickness range for maximizing luminance and how composition plays a critical role in determining luminance performance.

In Figure 5a, the luminance peaks at approximately 30–55 nm, suggesting that MoO_3_ offers a relatively high luminance for optimal hole injection. Beyond this range, luminance rapidly declines, showing that excessive thickness impairs hole injection efficiency. Figure 5b’s graph of HTL materials shows a broader luminance peak around 55–70 nm. This suggests that TAPC offers a relatively wider operational range for optimal hole transport. In the third graph, Figure 5c, usage of 3TPYMB as the ETL exhibits a luminance peak at around 45–50 nm thickness. The combined analysis proves each layer has a specific thickness range where the device achieves maximum luminance, highlighting the need for precise thickness control to refine OLED efficiency, ensuring a high brightness and stable light output.

#### 3.1.2. The Effect of Layer Thickness on Color Coordinates

In this step, we used SETFOS simulation software for the OLED stack in Figure 1, exchanging and utilizing the combination of candidate materials shown in Table 2.

**Figure 6:**
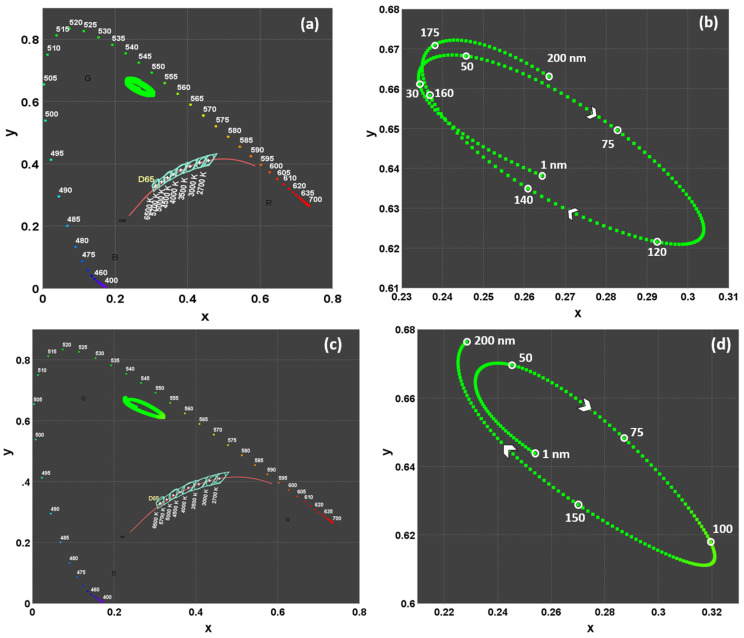

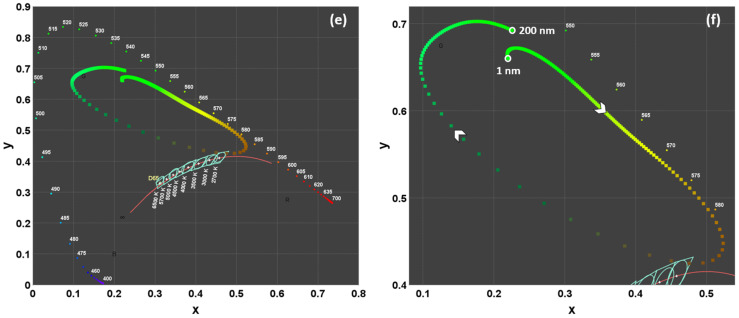
Color coordinate variations for 1–200 nm thickness ranges of (**a**) MoO_3_; (**b**) MoO_3_, zoomed for 0.08 < u′ < 0.14; (**c**) TAPC; (**d**) TAPC, zoomed for 0.08 < u′ < 0.13; (**e**) 3TPYMB; and (**f**) 3TPYMB, zoomed for 0.03 < u′ < 0.23, all in CIE 1931. (R, G, B, means Green, Red, Blue Zones).

Figure 6 shows the variation in CIE 1931 color coordinates with respect to the thickness of the best HIL/HTL/ETL material triad, detected as MoO_3_/TAPC/3TPYMB. The plotted data points trace an elliptical pattern, indicating how the color coordinates change as the HIL/HTL/ETL thickness varies from 1 to 200 nm. MoO_3_ (as HIL) and TAPC (as HTL) thicknesses have a moderate yet measurable impact on the color properties of the NVIS-OLED stack (Figure 6a,b). However, 3TPYMB (as ETL) thickness has a significant impact on color coordinates as seen in Figure 6c.

**Figure 7:**
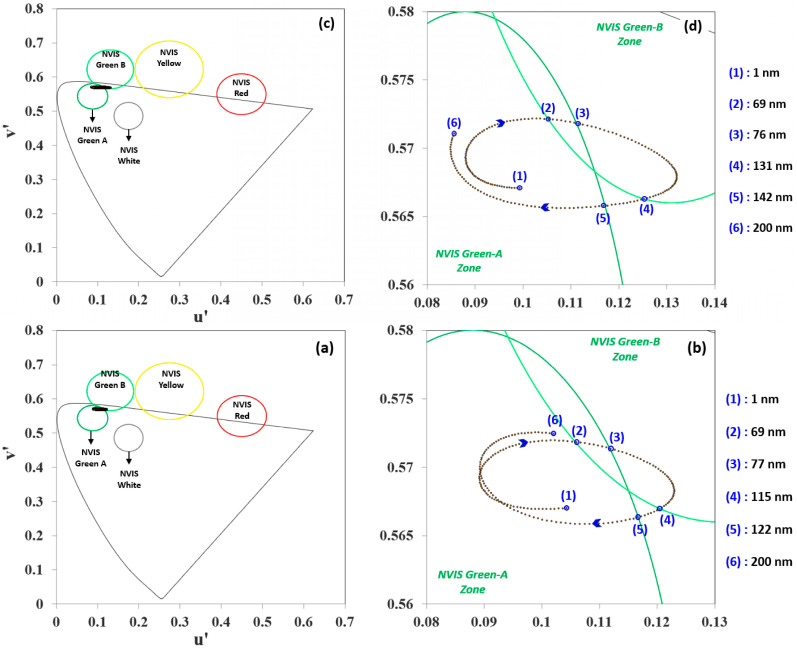

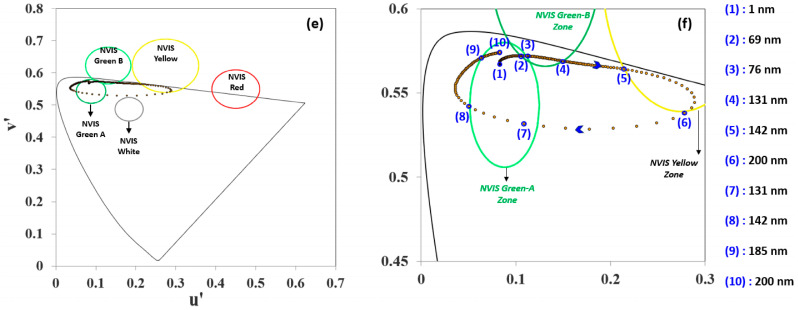
Color coordinate variations for 1–200 nm thickness ranges of (**a**) MoO_3_; (**b**) MoO_3_, zoomed for 0.08 < u′ < 0.13; (**c**) TAPC; (**d**) TAPC, zoomed for 0.08 < u′ < 0.14; (**e**) 3TPYMB; and (**f**) 3TPYMB, zoomed for 0 < u′ < 0.3, all in CIE 1976.

Since the NVIS colors are originally represented in the u′-v′ system within CIE 1976, we need a representation like in Figure 7 that plots the data of Figure 6 for the CIE 1976 system instead of CIE 1931. As the thickness grows, the color coordinates traverse and cross the boundaries of the NVIS Green A and NVIS Green B regions in an elliptical manner. In the MoO_3_ and TAPC cases, upon a 1 nm step increase in ETL thickness within the 1–200 nm range, chromaticity variation covers only the NVIS Green A and NVIS Green B regions; whereas, in the 3TPYMB case, the chromaticity value of the emitted light from the EML orderly traverses the NVIS Green A, NVIS Green B, and NVIS Yellow regions in a wider elliptical loop. The ETL thickness is thus observed to affect the color more than the other HIL and HTL thicknesses. 

#### 3.1.3. Effect of the Reflectivities of the Layer Materials on Luminance and Color Coordinates

**Figure 8:**
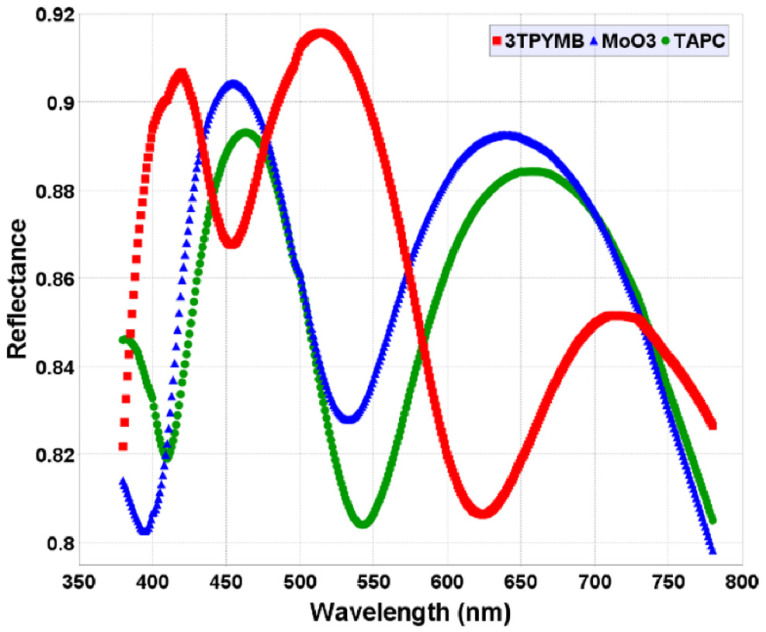
Reflectance variation with respect to MoO_3_;, TAPC, and 3TPYMB thicknesses (1–100 nm).

Figure 8 exhibits the reflectance of MoO_3_, TAPC, and 3TPYMB as a function of the wavelength in the visible spectrum, ranging from approximately 375 to 775 nm.

The plot reveals peaks and troughs, indicating specific wavelengths where the reflectance is locally highest or lowest. These oscillations suggest constructive and destructive interference effects stemming from the reflection calculated through Equations (S1)–(S10) in Supplementary File S1 at visible spectrum wavelengths.

**Figure 9:**
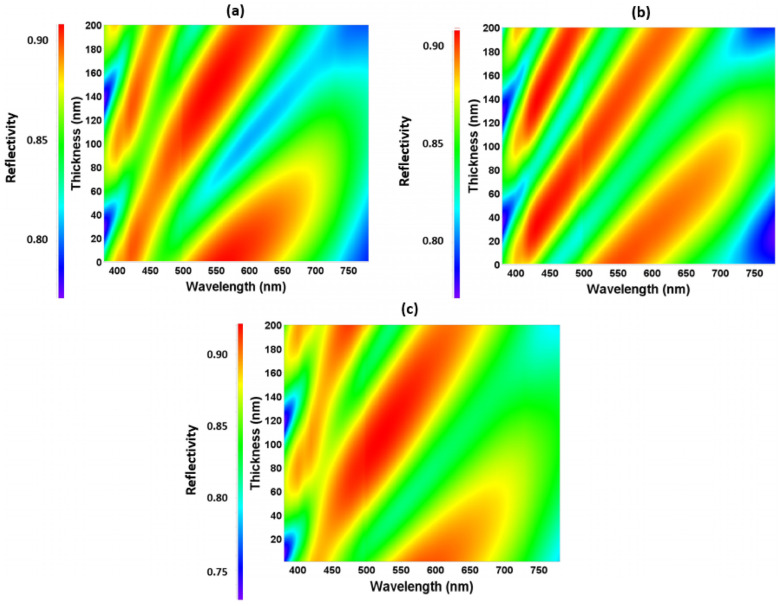
Color coordinate variation with respect to (**a**) MoO_3_, (**b**) TAPC, and (**c**) 3TPYMB thicknesses.

The heatmaps in Figure 9 provide a visual representation of how reflectance varies with both wavelength (x-axis) and MoO_3_, TAPC, and 3TPYMB thicknesses (y-axis, 1–200 nm). Figure 8 is a 2-D representation, whereas Figure 9 can be imagined as a 3-D representation of same data. The color gradient from blue (low reflectance) to red (high reflectance) shows the reflectance behavior across the thickness range of 1–200 nm and within the visual wavelengths range. The color bands highlight the regions of high and low reflectance and how varying the MoO_3_, TAPC, and 3TPYMB thicknesses impacts the optical response. This thickness-dependent modulation of reflectance can be attributed to the interference effects within the respective thin film layer, affecting how light is reflected from the same. Adjusting the MoO_3_, TAPC, and 3TPYMB thicknesses allowed for the fine-tuning of reflectance at the desired wavelengths, improving device efficiency by minimizing unwanted reflections or enhancing desired optical characteristics.

**Figure 10:**
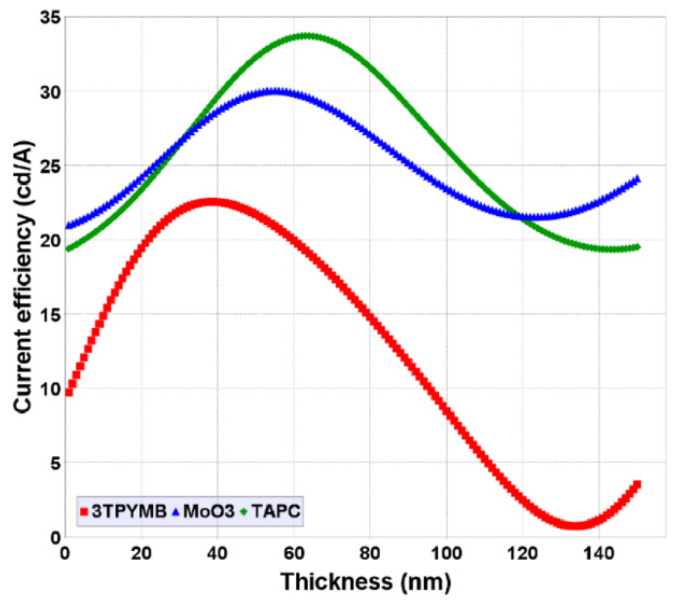
Color coordinate variation with respect to HIL, HTL, and ETL thicknesses (1–150 nm).

Figure 10 shows the color coordinate variations with respect to the HIL, HTL, and ETL thicknesses (1–150 nm). At thicknesses between 60 and 80 nm, the current efficiency and luminance values increased to maximum values, thus we observed how the high reflectance of the ETL-EIL intralayer lead to lower overall optical efficiency of the OLED device. 

### 3.2. Model Validation

**Figure 11:**
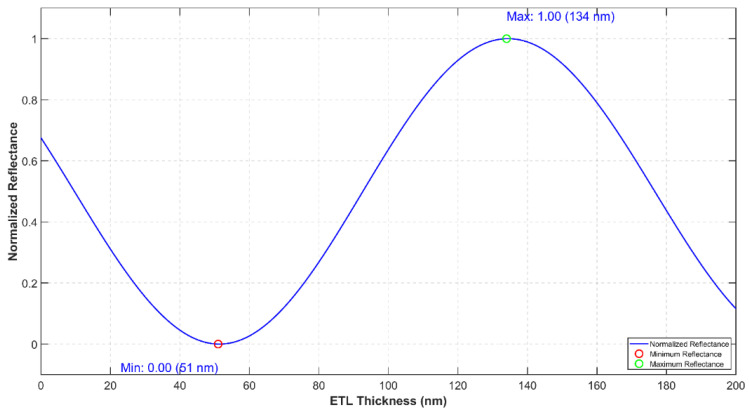
Normalized reflectance (from cathode) as a function of ETL (3TPYMB) thickness in Glass/ITO/MoO_3_/TAPC/(CBP:Ir(ppy)_3_)/3TPYMB/LiF/Al OLED stack architecture (MATLAB output).

This section utilizes the theoretical framework established in the previous sections to examine specific OLED layers. Figure 11 illustrates the relationship between reflectance and the thickness of the electron transport layer (ETL). A thorough understanding of the refractive index (n) and extinction coefficient (k) of the OLED layers is crucial for grasping the behavior of light as it propagates through and reflects within the device. In Table 5, the values for each material within the OLED configuration are provided, accompanied by a discussion of their significance in influencing reflectance. The required theory and equations for this chapter are presented in Supplementary File S1—Effective Reflectivity Calculation.

For the glass/ITO/MoO_3_/TAPC/(CBP:Ir(ppy)_3_)/3TPYMB/LiF/Al configuration, the impact of both constructive and destructive interference on the system is highlighted in Figure 11, which illustrates how reflectance varies with the thickness of the electron transport layer (ETL). Figure 11 shows the best and worst ETL thicknesses for the chosen materials. Based on the provided figure, it is anticipated that the lowest luminous intensity will be measured at an ETL thickness of 51 nm, and the highest luminous intensity will be measured at a thickness of 134 nm.

### 3.3. Model Validation Using LightTools

**Figure 12:**
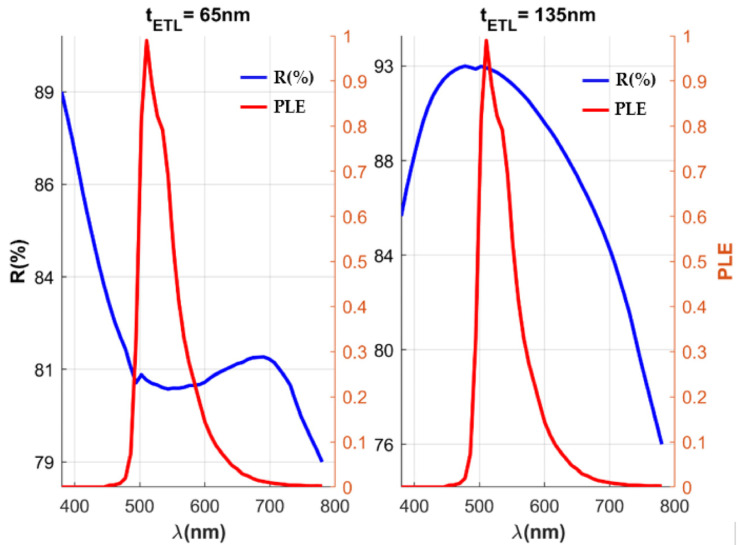
Photoluminescent Emission (PLE) spectrum (normalized) versus % reflectance (R) as a function of wavelength for ETL thicknesses of 65 nm and 135 nm (LightTools). (All simulation results have been given in Figure S1 in Supplementary File S2).

The OLED architecture in the simulation environment is split into two sections referred to as the “top stack” and the “bottom stack” (Figure 2). As our transmittance has its peak at 511 nm for our EML layer, to maximize optical efficiency, we need to optimize for maximum transmittance in the bottom stack and the lowest reflectance in the EIL-ETL layers of the top stack at this wavelength. Upon analyzing, we observed that when ETL (CBP) thickness is around 65 +/− 10 nm the ETL-EIL reflectance values at 511 nm were minimum and when the ETL (CBP) thickness is around 135 +/− 10 nm the reflectance values at 511 nm were maximum (Figure 12).

**Figure 13:**
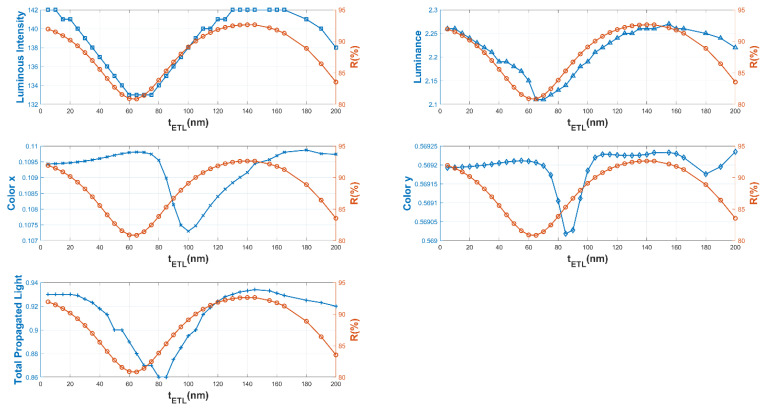
ETL thickness and resulting cathode reflectance versus the luminance, color coordinates, total light propagation, and luminous intensity for Glass/ITO/MoO_3_/CBP/CBP:Ir(ppy)_3_/3TPYMB/LiF/Al OLED stack architecture (LightTools).

For the glass/ITO/MoO_3_/CBP/CBP:Ir(ppy)_3_/3TPYMB/LiF/Al configuration, the highest luminous intensity is measured at an ETL thickness of 67 nm, and the lowest luminous intensity is produced at a thickness of 134 nm as shown in Figure 13.

Table 6 illustrates the effects of layer material and layer thickness on chromaticity shifts, represented by % Δu′ and % Δv′, in an optoelectronic device. Among the materials, the electron transport layer (ETL) has the highest impact, with % Δu′ at 22.03% and % Δv′ at 0.23%, indicating its critical role in color stability, while the hole transport layer (HTL) and hole injection layer (HIL) show relatively minor effects. In contrast, layer thickness significantly influences chromaticity, with the ETL exhibiting the largest shifts at 139.73% for % Δu′ and 5.74% for % Δv′, followed by substantial impacts from the HTL and HIL. These findings emphasize the importance of optimizing both material selection and thickness, particularly for the ETL, to achieve the desired chromaticity and device performance.

**Figure 14:**
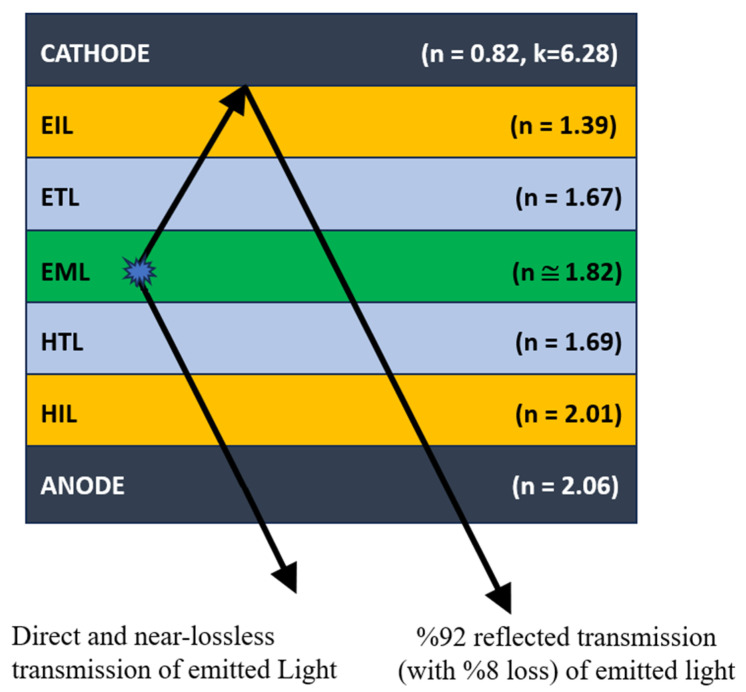
Light emitted from EML (through ETL-EIL) bounces back from cathode (with %92 reflectivity) when ETL thickness is optimized.

After being produced within the EML, the light rays choose two main paths, towards the cathode and towards the anode. Since we propose a bottom emitting OLED, exit from the anode is acceptable, but reflections from the cathode should be maximized with minimal interlayer (EIL, ETL) loss so that light efficiency is enhanced. We depicted this by updated Figure 14.

To enhance optical efficiency, we anticipate elevated transmittance in the layer referred to as the “bottom stack” and in the layer designated as the “top stack”. The produced light can traverse the cathode layer without loss and be reflected back from the cathode layer to the regaining systems. These methods enhance optical stack efficiency.

Table 7 provides evidence and a summary of the 35 simulations performed in SETFOS^®^ on how the effectiveness of an optoelectronic device varies with the options of layer materials, their thicknesses, and the so-resulting reflectivity (R) of the cathode in Figure 14. A high brightness (3470 cd/m^2^) and luminous efficacy (34.73 cd/A) is achieved where the cathode reflectivity forms at 92%, in addition to the use of MoO_3_; (55 nm) as the HIL, TAPC (63 nm) as the HTL, and 3TPYMB (48 nm) as the ETL. The average performing device had an 85.36% reflectivity with CBP (50 nm) and TPBi (40 nm) as the HIL and ETL, respectively, and this results in the depreciation of luminescence and its efficiency. Lowest performance was defined by achieving a 60% reflectivity from the cathode together with CuPc (84 nm) as the HIL and TPBi and CBP, which produced the poorest brightness and efficiency of 1515 cd/m^2^ and 15.15 cd/A, respectively.

## 4. Conclusions

In this manuscript, the thin film thickness control in an NVIS-OLED display application is demonstrated and the sensitivity of color coordinates to changes in the ETL materials underlined the criticality of material selection for the ETL to achieve the desired color properties especially for NVIS compatibility. By simulations, it is proven to be possible that the thickness optimization of ETL material could enhance luminance by reducing interlayer reflectance within the OLED device. This optimization should be made for the layers between the EML and the reflective cathode especially. The impact on the key performance parameters was analyzed using SETFOS and LightTools as the device simulation tools. Within the 35 different device combinations, an OLED architecture of the Glass/ITO/MoO_3_/TAPC/CBP:Ir(ppy)_3_/3TPYMB/LiF/Aluminum combination showed promise, with better luminance up to 23.8%, when cathode reflectance through the EIL-ETL layer is enhanced only %5.1 (from %87.5 to %92) with respect to a suboptimal OLED device with an exact material stack. It became apparent that adverse outcoupling effects can be minimized when the ETL-EIL interlayer transmissivities are maximized. We conclude that our findings may offer valuable insights for research efforts concerning military OLED displays. The biggest impact of this manuscript is that these comprehensive investigations are performed for the Night Vision Compatibility of OLED devices for the first time.

## Figures and Tables

**Table 1:** NVIS MIL-STD-3009 chromaticity limitations for CIE 1976 color system.

**Color**	u′	v′	r
NVIS Green A	0.088	0.543	0.037
NVIS Green B	0.131	0.623	0.057
NVIS Yellow	0.274	0.622	0.083
NVIS Red	0.450	0.550	0.060
NVIS White	0.190	0.490	0.040

**Table 2:** The materials list of simulated OLED devices.

ANODE	HIL	HTL	EML	ETL	EIL	CATHODE
ITO	MoO_3_	NPB	CBP:Ir(ppy)_3_	3TPYMB	LiF	Al
	CuPc	TAPC		BCP		
	Alfa-NPD	Tris-PCz		BPhen		
	HATCN	TCTA		TPBi		
		mCP		TMPYPB		
		mCBP		Balq		
		CBP		B3PYMPM		
		m-MTDATA		B4PYMPM		
		SpiroOMeTAD		Alq_3_		
		2-TNATA		Alq		

**Table 3:** A list of material abbreviations.

Abbreviation	Full Name
ITO	Indium Tin Oxide
MoO_3_	Molybdenum Trioxide
CuPc	Copper Phthalocyanine
α-NPD	N,N′-Bis(naphthalen-1-yl)-N,N′-bis(phenyl)benzidine
HATCN	1,4,5,8,9,11-Hexaazatriphenylenehexacarbonitrile
TAPC	1,1-Bis[4-(di-p-tolylamino) phenyl] cyclohexane
TCTA	Tris(4-(9H-carbazol-9-yl) phenyl) amine
TrisPCZ	Tris(9-phenylcarbazol-3-yl) benzene
NPB	N,N′-Bis(naphthalen-1-yl)-N,N′-bis(phenyl)benzidine
mCBP	3,3′-Bis(N-carbazolyl)-1,1′-biphenyl
mCP	1,3-Bis(N-carbazolyl) benzene
m-MTDATA	4,4′,4″-Tris[3-methylphenyl(phenyl)amino] triphenylamine
SpiroOMeTAD	2,2′,7,7′-Tetrakis [N,N-di(4-methoxyphenyl) amino]-9,9′-spirobifluorene
CBP	4,4′-Bis(N-carbazolyl)-1,1′-biphenyl
2-TNATA	4,4′,4″-Tris(N-(naphthalen-2-yl)-N-phenylamino) triphenylamine
3TPYMB	Tris[3-(3-pyridyl) mesityl] borane
TPBi	1,3,5-Tris(N-phenylbenzimidazol-2-yl) benzene
BCP	2,9-Dimethyl-4,7-diphenyl-1,10-phenanthroline (Bathocuproine)
TMPYPB	Tris[4-(pyridin-3-yl) phenyl] amine
BPhen	4,7-Diphenyl-1,10-phenanthroline (Bathophenanthroline)
Balq	Bis(2-methyl-8-hydroxyquinolinato) aluminum (III)
Alq	-
B4PYMPM	Bis-4,6-(3,5-di(pyridin-3-yl) phenyl)-2-methylpyrimidine
B3PYMPM	Bis-4,6-(3-(pyridin-3-yl) phenyl)-2-methylpyrimidine
Alq_3_	Tris(8-hydroxyquinolinato) aluminum (III)
LiF	Lithium Fluoride
Al	Aluminum

**Table 4:** Different layer materials and resulting color coordinates of the OLED device.

Layer	Layer Material	u′	v′	NVIS Color
Hole Injection	MoO_3_	0.096153	0.571686	NVIS Green A
Hole Injection	CuPc	0.095147	0.570567	NVIS Green A
Hole Injection	α-NPD	0.097469	0.571293	NVIS Green A
Hole Injection	HATCN	0.097223	0.571321	NVIS Green A
Hole Transport	TAPC	0.100431	0.572124	NVIS Green A
Hole Transport	TCTA	0.099879	0.57195	NVIS Green A
Hole Transport	TrisPCZ	0.099345	0.571828	NVIS Green A
Hole Transport	NPB	0.098783	0.57184	NVIS Green A
Hole Transport	mCBP	0.100846	0.572048	NVIS Green A
Hole Transport	mCP	0.100661	0.572045	NVIS Green A
Hole Transport	m-MTDATA	0,100754	0.572046	NVIS Green A
Hole Transport	SpiroOMeTAD	0.102798	0.572224	NVIS Green A
Hole Transport	CBP	0.099512	0.571944	NVIS Green A
Hole Transport	2-TNATA	0.101059	0.572082	NVIS Green A
Electron Transport	3TPYMB	0.107328	0.57214	NVIS Green B
Electron Transport	TPBi	0.130216	0.570932	NVIS Green B
Electron Transport	BCP	0.105361	0.572182	NVIS Green A
Electron Transport	3TMPYPB	0.107062	0.572188	NVIS Green B
Electron Transport	BPhen	0.105882	0.57218	NVIS Green A
Electron Transport	Balq	0.107074	0.572168	NVIS Green B
Electron Transport	B4PYMPM	0.10491	0.572227	NVIS Green A
Electron Transport	B3PYMPM	0.10491	0.572227	NVIS Green A
Electron Transport	Alq_3_	0.106045	0.572224	NVIS Green B
Electron Transport	Alq	0.105857	0.572221	NVIS Green B

**Table 5:** OLED layers and their optical properties. (Wavelength of Light: 511 nm).

Layer	Refractive Index (n)	Extinction Coefficient (k)	Thickness (nm)
Cathode (Aluminum)	0.826	6.28	100
Electron Injection Layer (LiF)	1.394095	0	1
Electron Transport Layer (3TPYMB)	1.6677	$4.20\;\times \;10^{-16}$	0–200
Emissive Layer (CBP:Ir(ppy)_3_)	1.8189	$1.27\;\times \;10^{-5}$	40

**Table 6:** Effect of layer material on variation of green color coordinates.

Effect	Layer	% Δu′	% Δv′
Layer Material	Hole Injection (HIL)	2.41	0.20
Layer Material	Hole Transport (HTL)	3.98	0.07
Layer Material	Electron Transport (ETL)	22.03	0.23
Layer Thickness	Hole Injection (HIL)	31.83	1.18
Layer Thickness	Hole Transport (HTL)	43.52	1.15
Layer Thickness	Electron Transport (ETL)	139.73	5.74

**Table 7:** Effect of layer materials and thicknesses on Cathode Reflectivity, Luminance (L_v_), Current Efficiency (CE) performance, color coordinates, and %EQE.

Performance	HIL	HTL	ETL	%R	L_v_ (cd/m^2^)	CE (cd/A)	u′	v′	EQE (%)
Best (15 *)	MoO_3_ (55 nm)	TAPC (63 nm)	3TPYMB (51 nm)	92	3470	34.73	0.1109	0.5721	9.31
Average (2 *)	CuPc (35 nm)	CBP (50 nm)	TPBi (40 nm)	87.5	2800	28.05	0.0952	0.5706	7.93
Worst (26 *)	CuPc (84 nm)	CBP (50 nm)	TPBi (40 nm)	60	1515	15.15	0.1178	0.5708	4.27

*: simulation number in the 35-experiment database in Supplementary File S2.

## Data Availability

Data are available in the Supplementary Material.

## Supplementary Materials

The following supporting information can be downloaded at https://www.mdpi.com/article/10.3390/mi16020191/s1: File S1: Mathematical Background, File S2: All Simulation Results.
