# Supplementary material for: Impact of Layer Materials, Their Thicknesses, and Their Reflectivities on Emission Color and NVIS Compatibility in OLED Devices for Avionic Display Applications
Source: Micromachines (Basel). 2025 Feb 7;16(2):191. doi: 10.3390/mi16020191 (PMC11857542; doi:10.3390/mi16020191)
Supplement: Supplementary file 1 [file micromachines-16-00191-s001.zip › File S1.pdf]

SUPPLEMENT-S1 – Mathematical Background

Wave Propagation in Thin Films

Maxwell's equations can be used to describe the electric field associated with a plane electromagnetic wave in a multilayer thin-film system. The wave equation governs the electric field in each layer j of thickness d_j. It is expressed as follows:

$$E_{j(z)} = E_j^{+} e^{\{i\, k_j z\}} + E_j^{-} e^{\{-i\, k_j z\}} \tag{S1}$$

where the complex refractive index is $n_j = n_j^{\text{real}} - ik_j$, the wave vector in the medium is $k_j = \frac{2\pi n_j}{\lambda}$, the forward and backward traveling wave components are E_j^{+} and E_j^{-}, and the wavelength of light in vacuum is λ.

Characteristic Matrix for a Single Layer

A 2×2 characteristic matrix can be used to relate the electric field components at the layer boundaries for each layer j. The following represents the characteristic matrix of a layer j with thickness d_j and complex refractive index $n_j = n_j^{\text{real}} - ik_j$:

$$M_j = \begin{bmatrix} \cos(\delta_j) & \frac{i\sin(\delta_j)}{\eta_j} \\ i\eta_j \sin(\delta_j) & \cos(\delta_j) \end{bmatrix} \tag{S2}$$

where $\delta_j = \frac{2\pi n_j d_j}{\lambda}$ is the phase thickness, and $\eta_j = n_j$ is the characteristic impedance of the layer, assumed to be the complex refractive index.

Phase Shift Due to ETL Thickness

The phase shift (δ) caused by light reflecting from the bottom surface of the ETL depends on the thickness of the ETL layer and the wavelength of light. The phase shift is calculated as:

$$\delta = \frac{2\pi n_{ETL} d}{\lambda} \tag{S3}$$

where d is the thickness of the ETL layer, n_{ETL} is the refractive index of the ETL layer, and λ is the wavelength of the incident light. The wave mechanics gives rise to the factor 2π. A wave's complete cycle is represented by a phase shift of 2π radians. To express the phase difference in

terms of radians, the optical path difference $2n_{ETL}d$ is used to measure how much farther light must travel through the ETL layer. This is normalized by the wavelength λ.

Constructive and Destructive Interference

For thin films, constructive and destructive interference depend on the relationship between the optical path difference and the wavelength. These conditions are given as:

$$2n_{ETL}d\cos(\theta) = m\lambda \text{ (Constructive Interference)} \quad \text{(S4)}$$

$$2n_{ETL}d\cos(\theta) = \left(m + \frac{1}{2}\right)\lambda \text{ (Destructive Interference)} \quad \text{(S5)}$$

where m is the order of interference and θ is the angle of incidence. At normal incidence, $\cos(\theta) = 1$.

Impact of Thickness on Interference Patterns

As the thickness of the ETL layer changes, the optical path difference for light reflecting off the ETL layer also changes. This alters the phase shift (δ), which affects whether the reflected light waves interfere constructively or destructively.

Constructive Interference occurs when the phase shift leads to waves reinforcing each other, increasing reflectance. Destructive Interference occurs when the phase shift causes the reflected waves to cancel each other out, reducing reflectance.

As the ETL thickness changes, the alternating conditions for constructive and destructive interference repeat periodically. For certain thicknesses, the light waves reflect in phase (constructive interference), and for other thicknesses, the reflected waves are out of phase (destructive interference). This is why reflectance fluctuates as a function of ETL thickness.

From the interference condition, when the ETL thickness satisfies the destructive interference condition:

$$2n_{ETL}d = \left(m + \frac{1}{2}\right)\lambda \quad \text{(S6)}$$

The minimization of reflectance occurs due to the cancellation of reflected waves. Variations in the thickness of the ETL lead to alterations in the phase difference δ, which in turn modifies the interference pattern, transitioning between constructive and destructive interference.

Total Transfer Matrix for Multilayer Stack

The total transfer matrix M_{total} in a multilayer system with N layers is obtained by multiplying the characteristic matrices of the individual layers:

$$M_{\text{total}} = M_1 M_2 \ldots M_N \tag{S7}$$

In this context, we examine four distinct layers: the cathode, the electron injection layer (EIL), the electron transport layer (ETL), and the emissive layer (EML). The computational process initiates at the ETL layer, as light is incident from the EML layer. The complete matrix of transfers is as follows:

$$M_{\text{total}} = M_{\text{ETL}} M_{\text{EIL}} M_{\text{cathode}} \tag{S8}$$

Reflection Coefficient and Reflectance

The overall transfer matrix and the impedance mismatch between the incident medium (EML) and the exit medium (cathode) determine the reflection coefficient r at the interface between the Emissive Layer (EML) and the Electron Transport Layer (ETL). The following provides the reflection coefficient r:

$$r = \frac{M_{11} + M_{12}\eta_{\text{cathode}} - \eta_{\text{EML}}(M_{21} + M_{22}\eta_{\text{cathode}})}{M_{11} + M_{12}\eta_{\text{cathode}} + \eta_{\text{EML}}(M_{21} + M_{22}\eta_{\text{cathode}})} \tag{S9}$$

where η_{EML} is the characteristic impedance of the Emissive Layer, M_{11}, M_{12}, M_{21}, and M_{22} are elements of the total transfer matrix M_{total}, and the cathode's impedance is $\eta_{\text{cathode}} = n_{\text{cathode}} - ik_{\text{cathode}}$. The magnitude squared of the reflection coefficient is the reflectance R.

$$R = |r|^2 \tag{S10}$$

Results on R with respect to material thicknesses are shown in figures x,y,z.
