# Supplementary material for: Impact of Layer Materials, Their Thicknesses, and Their Reflectivities on Emission Color and NVIS Compatibility in OLED Devices for Avionic Display Applications
Source: Micromachines (Basel). 2025 Feb 7;16(2):191. doi: 10.3390/mi16020191 (PMC11857542; doi:10.3390/mi16020191)
Supplement: Supplementary file 1 [file micromachines-16-00191-s001.zip › File S2.pdf]

## SUPPLEMENT-S2 All Simulation Results

**Table S1.** Current Efficiency (CE), Luminance (Lv), Cathode Reflectance (R) for 35 simulation experiments with SETFOS (for current density through device of 10mA/cm$^2$)

| Exp Number | Experiment Name | %R | Lv (cd/m2) | CE (cd/A) |
|---|---|---|---|---|
| 1 | HIL_MoO3 | 90.5 | 3000 | 30.00 |
| **2** | **HIL_CuPc** | **87.5** | **2800** | **28.05** |
| 3 | HIL_aNPD | 90 | 2910 | 29.10 |
| 4 | HIL_HATCN | 91 | 2913 | 29.13 |
| 5 | HTL_TAPC | 90 | 3368 | 33.68 |
| 6 | HTL_TCTA | 90.5 | 3003 | 30.03 |
| 7 | HTL_TrisPCZ | 91 | 2810 | 28.10 |
| 8 | HTL_NPB | 90 | 2850 | 28.50 |
| 9 | HTL_mCBP | 90.5 | 3185 | 31.85 |
| 10 | HTL_mCP | 90.5 | 3200 | 31.96 |
| 11 | HTL_mMDATA | 90 | 3095 | 30.95 |
| 12 | HTL_SpiroOMeTAD | 90 | 3288 | 32.88 |
| 13 | HTL_CBP | 90.5 | 3027 | 30.27 |
| 14 | HTL_2TNATA | 90.5 | 3078 | 30.78 |
| **15** | **ETL_3TPYMB** | **92** | **3470** | **34.73** |
| 16 | ETL_TPBi | 91 | 3188 | 31.88 |
| 17 | ETL_BCP | 91 | 3450 | 34.50 |
| 18 | ETL_TMPYPB | 91 | 3425 | 34.25 |
| 19 | ETL_BPHEN | 91 | 3440 | 34.40 |
| 20 | ETL_Balq | 91 | 3460 | 34.60 |
| 21 | ETL_B4PYMPM | 91 | 3400 | 34.00 |
| 22 | ETL_B3PYMPM | 91 | 3400 | 34.00 |
| 23 | ETL_Alq3 | 90 | 3435 | 34.35 |
| 24 | ETL_Alq | 90 | 3420 | 34.20 |
| 25 | HIL_CuPc_v2 | 62.5 | 1650 | 16.50 |
| **26** | **HIL_CuPc_v3** | **60** | **1515** | **15.15** |

| | | | | |
|---|---|---|---|---|
| 27 | HIL_MoO3_v2 | 83 | 2650 | 26.50 |
| 28 | HTL_TAPC_v2 | 80 | 3340 | 33.40 |
| 29 | HTL_SpiroOMeTAD_v2 | 76.5 | 3225 | 32.25 |
| 30 | HIL_CuPc_v4 | 68 | 1950 | 19.50 |
| 31 | HTL_TCTA_v2 | 82.5 | 2950 | 29.50 |
| 32 | HTL_mCBP | 81 | 3150 | 31.50 |
| 33 | HTL_SpiroOMeTAD_v3 | 77.5 | 3025 | 30.25 |
| 34 | HTL_SpiroOMeTAD_v4 | 75 | 2250 | 22.5 |
| 35 | HIL_CuPc_v5 | 70 | 2090 | 20.9 |

## Results

**No.2:** Medium performance

**No.15:** Best performance

**No.26:** Worst performance

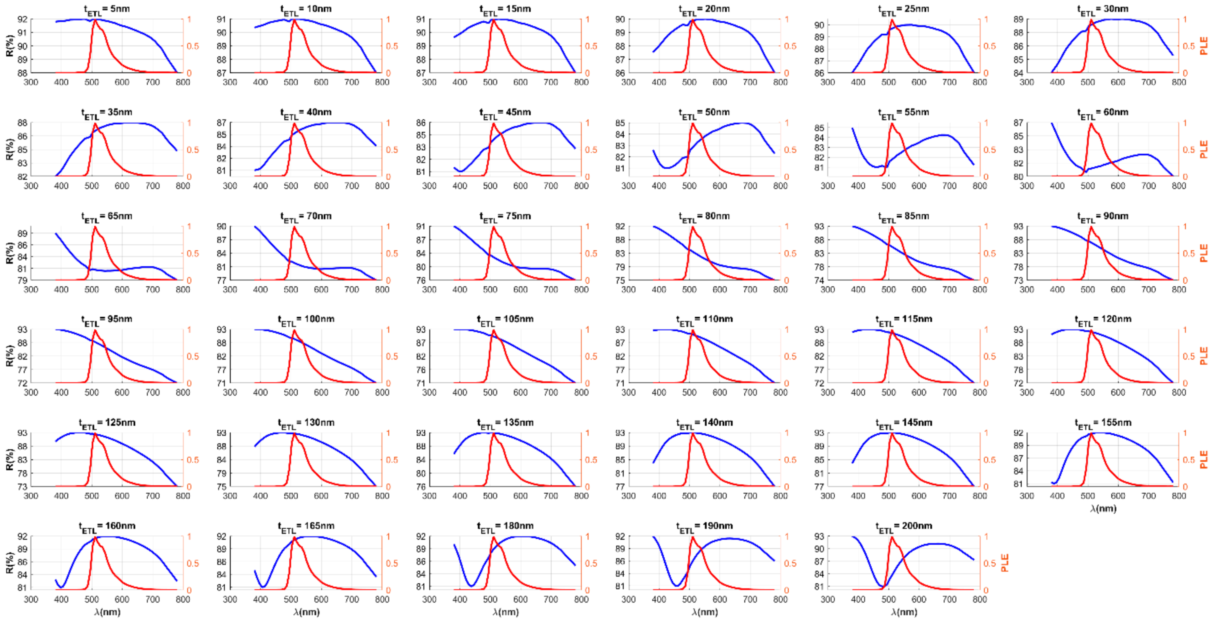

**Figure S1.** PL spectrum versus reflectance as a function of wavelength (LightTools)
